# College affirmative action bans and smoking and alcohol use among underrepresented minority adolescents in the United States: A difference-in-differences study

**DOI:** 10.1371/journal.pmed.1002821

**Published:** 2019-06-18

**Authors:** Atheendar S. Venkataramani, Erin Cook, Rourke L. O’Brien, Ichiro Kawachi, Anupam B. Jena, Alexander C. Tsai

**Affiliations:** 1 Department of Medical Ethics and Health Policy, Perelman School of Medicine, University of Pennsylvania, Philadelphia, Pennsylvania, United States of America; 2 Leonard Davis Institute of Health Economics, University of Pennsylvania, Philadelphia, Pennsylvania, United States of America; 3 Analysis Group, Boston, Massachusetts, United States of America; 4 La Follette School of Public Affairs, University of Wisconsin–Madison, Madison, Wisconsin, United States of America; 5 Department of Social and Behavioral Science, Harvard T.H. Chan School of Public Health, Boston, Massachusetts, United States of America; 6 Department of Health Care Policy, Harvard Medical School, Boston, Massachusetts, United States of America; 7 Chester M. Pierce, M.D. Division of Global Psychiatry, Massachusetts General Hospital, Boston, Massachusetts, United States of America; Harvard Medical School, UNITED STATES

## Abstract

**Background:**

College affirmative action programs seek to expand socioeconomic opportunities for underrepresented minorities. Between 1996 and 2013, 9 US states—including California, Texas, and Michigan—banned race-based affirmative action in college admissions. Because economic opportunity is known to motivate health behavior, banning affirmative action policies may have important adverse spillover effects on health risk behaviors. We used a quasi-experimental research design to evaluate the association between college affirmative action bans and health risk behaviors among underrepresented minority (Black, Hispanic, and Native American) adolescents.

**Methods and findings:**

We conducted a difference-in-differences analysis using data from the 1991–2015 US national Youth Risk Behavior Survey (YRBS). We compared changes in self-reported cigarette smoking and alcohol use in the 30 days prior to survey among underrepresented minority 11th and 12th graders in states implementing college affirmative action bans (Arizona, California, Florida, Michigan, Nebraska, New Hampshire, Oklahoma, Texas, and Washington) versus outcomes among those residing in states not implementing bans (*n* = 35 control states). We also assessed whether underrepresented minority adults surveyed in the 1992–2015 Tobacco Use Supplement to the Current Population Survey (TUS-CPS) who were exposed to affirmative action bans during their late high school years continued to smoke cigarettes between the ages of 19 and 30 years. Models adjusted for individual demographic characteristics, state and year fixed effects, and state-specific secular trends. In the YRBS (*n* = 34,988 to 36,268, depending on the outcome), cigarette smoking in the past 30 days among underrepresented minority 11th–12th graders increased by 3.8 percentage points after exposure to an affirmative action ban (95% CI: 2.0, 5.7; *p <* 0.001). In addition, there were also apparent increases in past-30-day alcohol use, by 5.9 percentage points (95% CI: 0.3, 12.2; *p =* 0.041), and past-30-day binge drinking, by 3.5 percentage points (95% CI: −0.1, 7.2, *p =* 0.058), among underrepresented minority 11th–12th graders, though in both cases adjustment for multiple comparisons resulted in failure to reject the null hypothesis (adjusted *p =* 0.083 for both outcomes). Underrepresented minority adults in the TUS-CPS (*n* = 71,575) exposed to bans during their late high school years were also 1.8 percentage points more likely to report current smoking (95% CI: 0.1, 3.6; *p =* 0.037). Event study analyses revealed a discrete break for all health behaviors timed with policy discussion and implementation. No substantive or statistically significant effects were found for non-Hispanic White adolescents, and the findings were robust to a number of additional specification checks. The limitations of the study include the continued potential for residual confounding from unmeasured time-varying factors and the potential for recall bias due to the self-reported nature of the health risk behavior outcomes.

**Conclusions:**

In this study, we found evidence that some health risk behaviors increased among underrepresented minority adolescents after exposure to state-level college affirmative action bans. These findings suggest that social policies that shift socioeconomic opportunities could have meaningful population health consequences.

## Abstract 2

Atheendar Venkataramani and co-workers compare smoking and alcohol use among minority college students in US states with and without affirmative action bans.

## Author summary

**Why was this study done?:**

- Between 1996 and 2013, 9 US states banned race-based affirmative action in college admissions.
- Affirmative action bans may have adverse spillover effects on health behavior among students belonging to underrepresented minority groups (i.e., racial and ethnic minorities that are underrepresented in higher education) through several different pathways. However, the consequences of affirmative action bans for health risk behaviors have not been studied.

**What did the researchers do and find?:**

- Using a dataset of about 35,000 high school students in the US, we compared changes in self-reported cigarette smoking and alcohol use among underrepresented minority 11th and 12th graders in states implementing college admission affirmative action bans versus among those residing in states not implementing bans.
- We found increases in self-reported cigarette smoking among underrepresented minority 11th and 12th graders, coinciding with the years affirmative action bans were discussed, passed, and implemented. We also found statistically nonsignificant increases in alcohol use after affirmative action bans. No associations were found for non-Hispanic White students.
- In a separate study of over 71,000 underrepresented minority adults aged 19–30 years, we found that those who were 16 years old (the typical age of a typical high school student entering the 11th grade) at the time an affirmative action ban was in place were more likely to report current smoking.

**What do these findings mean?:**

- This study finds that state-level affirmative action bans may result in increases in short- and long-term health risk behaviors among underrepresented minority students.
- The findings demonstrate the potential importance of social policies, particularly those that shift socioeconomic opportunities, for population health.

## Introduction

Socioeconomic factors have long been recognized as critical determinants of individual and population health [1–4]. In this vein, recent work has demonstrated a robust link between access to economic opportunities, health behaviors, and health outcomes [5–9]. Public policies that influence socioeconomic opportunities may have profound effects on health risk behaviors among adolescents [10], particularly those belonging to underrepresented minority groups (i.e., racial and ethnic minorities that are underrepresented in higher education), who face both elevated risks of morbidity and mortality [11] and restricted prospects for upward economic mobility [12].

**Fig 1:**
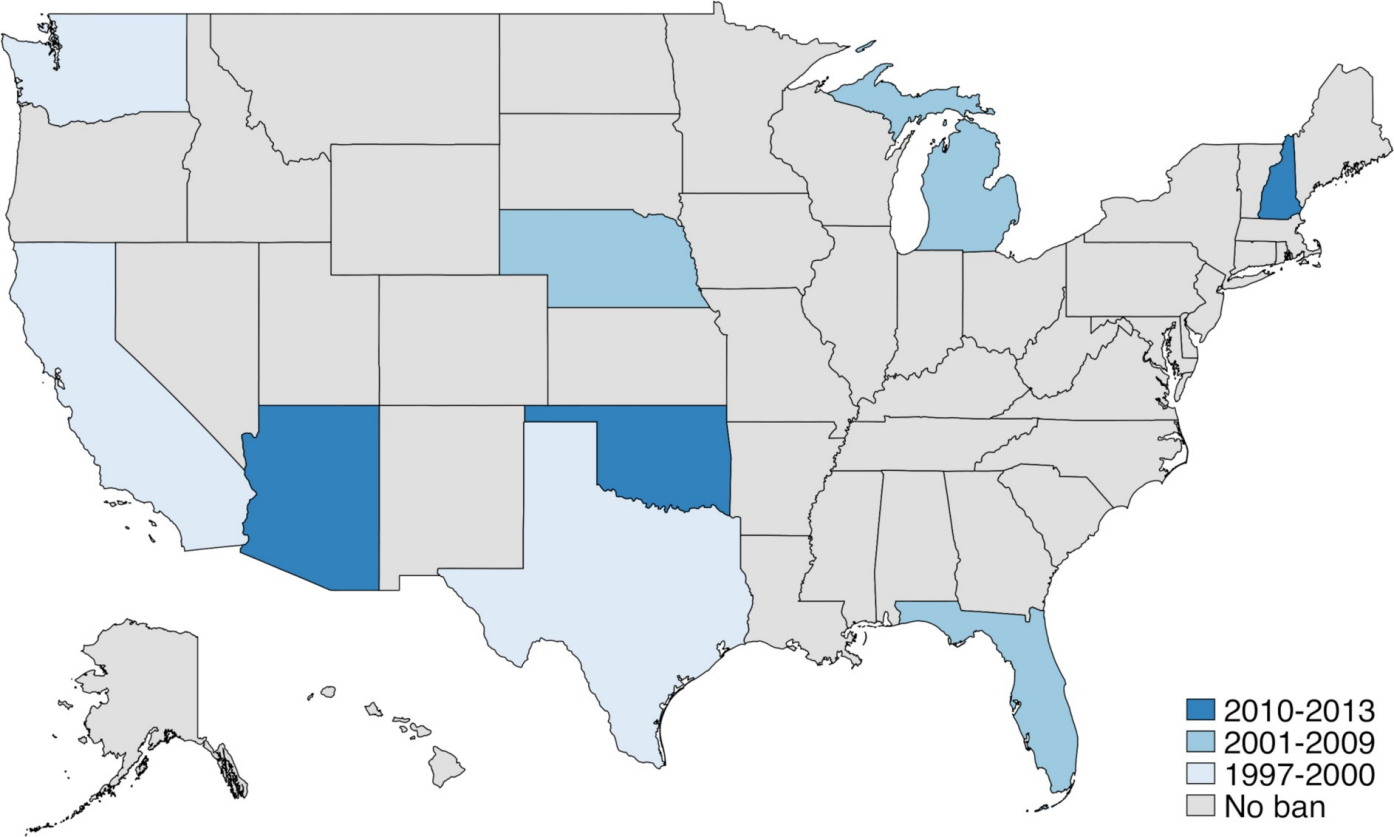
States implementing college affirmative action bans by year of implementation. Year of ban implementation: Texas—1997; California—1998; Washington—1999; Florida—2001; Michigan—2006; Nebraska—2008; Arizona—2010; New Hampshire—2012; Oklahoma—2013. See S1 Table for further details, including dates of court decisions and votes authorizing bans.

In the US, affirmative action policies have been used to directly remediate structural inequalities that have contributed to depressed socioeconomic outcomes among underrepresented minorities. The most well-known among these are programs that seek to enhance access to educational opportunities by incorporating race and ethnicity into college admission decisions. Some perceive that these programs unfairly disadvantage non-beneficiaries, resulting in significant political controversy. Along these lines, between 1996 and 2013, 9 states banned race-based affirmative action in college admissions (Fig 1). Currently, race-based affirmative action programs at several universities are facing high-profile legal challenges initiated by private parties and by the US Department of Justice [13–15].

A growing body of research has shown that US college affirmative action bans have reduced admission and graduation rates among underrepresented minorities at selective colleges [16–20]. In addition to these well-known impacts on educational outcomes, affirmative action bans may also have spillover consequences on health behaviors, particularly among high school students who are contemplating college. Active consideration and implementation of affirmative action bans may undermine underrepresented minority adolescents’ expectations of future economic opportunities, reducing their incentives to engage in positive health behaviors and/or avoid health risk behaviors [5,8,9]. Bans may communicate to underrepresented minority adolescents broader signals about aspects of the social environment, such as the degree of structural racism or societal discrimination, that are themselves associated with adverse health consequences [21–23]. Affirmative action bans may also increase competition for limited college admission slots, which could have mixed effects for underrepresented minority adolescents. On the one hand, bans could induce students to attempt to maximize their admission probabilities [24] by engaging in fewer health risk behaviors. On the other hand, bans may intensify exposure to academic stress [25,26] or demoralize adolescents faced with competition they perceive to be insurmountable [27,28], either of which could increase their risk of engaging in health risk behaviors. Adolescent health risk behavior may be particularly sensitive to these mechanisms, given that adolescence is a critical developmental period for forming, and acting on, beliefs about society and about the future [29–31].

These theoretically motivated hypotheses notwithstanding, the impacts of affirmative action bans on health risk behaviors are as of yet unknown. In this study, we estimated the association between race-based affirmative action bans and cigarette smoking and alcohol use among underrepresented minority adolescents. We used a quasi-experimental difference-in-differences design to estimate the change in outcomes before versus after policy implementation among underrepresented minority adolescents residing in states implementing affirmative action bans versus those residing in unaffected states. We also examined whether the association between exposure to an affirmative action ban and smoking persisted into adulthood.

## Methods

Institutional review board approval for this study was not required per University of Pennsylvania policy given the use of publicly available, deidentified data. This study did not have a prespecified analysis plan, but specification of all outcomes and exposures, the estimation sample, and statistical analyses were based on ex ante hypotheses (see S1 Appendix for further details). The datasets and code used for this project are posted at Harvard Dataverse (https://doi.org/10.7910/DVN/J7SOGC).

### Data and sample

We used data from the 1991–2015 US Youth Risk Behavior Survey (YRBS), a nationally representative repeated cross-sectional survey of 9th–12th graders in public and private schools fielded by the US Centers for Disease Control and Prevention (CDC) as part of the national Youth Risk Behavior Surveillance System (YRBSS). Surveys have been conducted biennially since 1991, typically in the spring. Survey participants are identified through a 3-stage sampling procedure, with oversampling of Black and Hispanic students at each stage [32]. The data include information on state of residence, individual demographic characteristics (age, sex, and race), and self-reported health risk behaviors. The national YRBS includes coverage—continuously before and after affirmative action policy changes—of data from 7 of the 9 states that implemented affirmative action bans during the study period, including the 2 largest states (Texas and California). (The 2 remaining states, Nebraska and New Hampshire, were both surveyed during a single wave, prior to implementation of bans.)

Estimation for this study focused on underrepresented minority students, defined as those who self-reported their race as “Black” or who self-reported their ethnicity as “Hispanic” or “Native American.” Given the role of affirmative action in remediating historically and structurally ingrained racial inequalities, we hypothesized that non-Hispanic White students would be differently affected by affirmative action bans, if at all. We did not analyze data on Asian-American and Pacific Islander students because their sample sizes were too small for robust inference. At the same time, Asian-American and Pacific Islander individuals as a group are not typically considered, as a matter of policy, to be underrepresented in higher education; therefore, it is unclear how changes to affirmative action policies may affect this group [33–35]. We restricted the sample to 11th and 12th grade students to focus on the distinct developmental stage of late high school, when decisions about college and future careers are particularly salient [36,37]. Observations with missing data were dropped from the analysis.

To examine the potential persistence of any estimated impacts of affirmative action bans on cigarette smoking into adulthood, we used data from the 1992–2015 Tobacco Use Supplement to the Current Population Survey (TUS-CPS) [38]. The TUS-CPS is a nationally and state-representative repeated cross-sectional survey of the US general population administered annually since 1992. These data include detailed information on current and past tobacco use. We focused on the same cohorts as in the YRBS—individuals belonging to underrepresented racial and ethnic minority groups who had attained the typical age of a high school junior between 1991 and 2015 (i.e., individuals who attained 16 years of age at any time point between 1990 and 2015). We further restricted the TUS-CPS sample to those aged 19–30 years at the time of the survey, so as to focus on young adults who had (likely) already exited high school and who were plausibly at risk of having been exposed to affirmative action bans during high school.

In both the YRBS and TUS-CPS, we excluded individuals residing in 4 states in which there was extended, multi-year litigation around affirmative action during the study period, but where bans were not actually implemented (Alabama, Georgia, Louisiana, and Mississippi). This exclusion follows from prior work on the educational consequences of affirmative action bans [18,19].

Further details on both datasets are provided in S1 Appendix.

### Outcomes and exposure

The primary outcomes in the YRBS were any self-reported cigarette smoking, alcohol use, and binge drinking in the 30 days prior to survey. We constructed binary measures for each outcome, using a threshold of at least 1 day or more of use. Using the TUS-CPS, we constructed a binary measure for current cigarette smoking, where individuals who reported smoking either “some days” or “every day” at the time of survey (versus “not at all”) were coded as current smokers. (See S1 Appendix for further details.)

The exposure of interest was a binary indicator indicating the implementation of an affirmative action ban in the respondent’s state of residence by the year the individual was in the 11th or 12th grade. YRBS respondents were considered exposed if an affirmative action ban had been implemented in the year of survey. For the sake of consistency with the YRBS analysis, TUS-CPS respondents were considered exposed if an affirmative action ban was in place in their state of residence during the calendar year they turned 16 years old, an age threshold that approximates the typical age of an 11th grader in high school.

Exposure assignment based on survey year (YRBS) or the calendar year the individual turned 16 years old (TUS-CPS) permitted exposures to occur just before actual ban implementation, which helps account for any potential shifts in future expectations due to rising media coverage of affirmative action bans in the period immediately prior to implementation (S1 Fig). (In the YRBS, surveys were typically conducted in the spring, while affirmative action bans were mostly implemented in the fall—see S1 Table. In the TUS-CPS, the autumn timing of enactment of most bans implies that the majority of individuals assigned as exposed would have turned 16 years of age prior to affirmative action ban implementation.) An important consideration specific to the TUS-CPS analysis is that, due to the longer lag between (presumed) exposure and the time of the survey, exposure assignment might also reflect any mediating role of post-high-school variables, such as college education or labor market outcomes.

### Statistical analysis

We used a quasi-experimental difference-in-differences design [39,40] to estimate the change in outcomes before versus after exposure to an affirmative action ban among underrepresented minority respondents residing in affected states versus those residing in unaffected states. (The estimating equation representing the regression model fitted to the data is provided in S1 Appendix.) We adjusted for age, sex, and race/ethnicity (Black, Hispanic, or Native American); state fixed effects, to account for time-invariant, state-level differences in socioeconomic, cultural, and political characteristics that could be correlated with affirmative action ban adoption and with the outcomes; racial/ethnic group–year (year of survey in the YRBS and year when the individual turned 16 years old in the TUS-CPS), to account for race/ethnic-group-specific national trends in the outcomes that could be coincident with affirmative action policy adoption; and state-specific linear time trends (again, specific to survey year in the YRBS or year the individual turned 16 years old in the TUS-CPS), to account for unobserved differential trends that jointly influence ban adoption and the outcomes. In the YRBS regression models, we additionally adjusted for 11th versus 12th grade status. In the TUS-CPS models, we additionally included fixed effects for year and month the survey was conducted (i.e., when the adult smoking outcomes were assessed).

We fitted all models using least squares, given well-known biases resulting from fitting fixed effects regression models with limited dependent variables [41]. In addition to estimating models for the pooled sample of underrepresented minority individuals, we also estimated models stratifying by sex and race/ethnicity (Black versus Hispanic), given potential differences in responses to stressful life events across these groups [42–44].

The 2 causal identification assumptions underlying the method of difference in differences are (1) no differential preexisting trends in the outcomes between exposed and unexposed states (the parallel trends assumption) and (2) no confounding from unmeasured state–year factors that may jointly be correlated with the exposure and outcome [39]. In addition to adjusting for state, year, and state-specific time trends as described above, we probed the validity of these assumptions by fitting event study models [45], in which we replaced the main exposure term with a series of binary variables denoting leads and lags of affirmative action bans ranging from 7 or more years before ban implementation to 6 or more years after (see S1 Appendix for the estimating equation). Individuals not residing in an affected state were coded as 0 for each of these variables. The event study approach provides a means to investigate violations of the parallel trends assumption underlying the validity of the difference-in-differences design. It also serves as a means to investigate the timing of health behavior changes—if these occur at the same time as the start of the exposure period, the role of unmeasured confounders can be considered less likely.

To additionally probe the underlying causal identification assumptions, we also estimated all models for non-Hispanic White individuals as a prespecified falsification test [46]. This procedure provided us with an opportunity to repeat the analysis under conditions expected to produce a null result [47] (because affirmative action bans would be unlikely to increase cigarette and alcohol use in this group). A null result observed among non-Hispanic White individuals would increase our confidence that the estimated effects among underrepresented minority individuals could be interpreted with the sociologically and historically specific meaning motivating our analysis.

For all models, we employed cluster-correlated robust standard errors to adjust confidence intervals and *p*-values for serial correlation in outcomes at the level of the state [48]. For the main difference-in-differences models using YRBS data, in which we examined 3 main outcomes, we additionally accounted for multiple comparisons by using the Sidak–Holm step-down method to compute *p-*values for each outcome that adjust for the family-wise error rate [49,50]. We conducted this procedure separately for the underrepresented minority student sample and the non-Hispanic White student sample, given our hypothesis that these groups would be differently affected by affirmative action bans [51]. (See S1 Appendix for further details.)

We used sampling weights to account for the complex survey designs when calculating descriptive statistics for the YRBS and TUS-CPS. However, we did not use weights in the regression models because the weighted least squares approach is known to be inefficient in settings where individual-level error terms are clustered within larger units (namely states, the unit of policy variation) [52]. (See S1 Appendix and S8 Table for further details.)

### Sensitivity analyses

We assessed the sensitivity of our findings to several alternative specifications, many of which were designed to further probe the underlying causal assumptions of the difference-in-differences method. First, we additionally adjusted for state- and year-specific cigarette tax rates, alcohol tax rates, (logarithm of) per capita income, and unemployment rates, all of which have been shown to influence health behaviors among adolescents [5,53,54]. Second, we accounted for potential geographic spillover effects of affirmative action bans by including in our models a separate binary indicator denoting exposure for adolescents in states adjacent to those implementing affirmative action bans [55]. Third, we reclassified as unexposed adolescents living in Texas in 2003 and thereafter, because some universities reestablished affirmative action programs after a favorable court ruling in 2003 [55]. (In 2019, the state of Washington repealed its 20-year affirmative action ban, but this policy change occurred outside the time frame of our study.) Fourth, we restricted estimation to respondents living in states that implemented an affirmative action ban at some point during the study period; in this analysis, these states serve as their own controls.

Fifth, in the TUS-CPS, we tested for an effect of a negative control exposure: first exposure to an affirmative action ban at the age of 19 years. Any measured association with this negative control exposure would suggest confounding by unobserved variables, given that college decisions are generally already made prior to this age.

Sixth, we used data from the Annual Social and Economic Supplement of the Current Population Survey (CPS-ASEC) [38]—a large, nationally and state-representative survey focused on socioeconomic outcomes that is conducted annually in March—to examine potential biases from nonrandom treatment assignment. Because the CPS-ASEC includes 16 to 18 year olds who are not in school, this analysis allowed us to examine the extent to which estimates using the school-based YRBS are biased (if at all) by differential school dropout in response to affirmative action bans. The CPS-ASEC also includes data on cross-state migration within the year prior to survey, allowing us to assess whether participants differentially migrated away from certain states subsequent to implementation of an affirmative action ban.

## Results

### Descriptive statistics

Table 1 provides descriptive statistics for underrepresented minority high school students residing in the states that implemented an affirmative action ban versus those in states that did not implement a ban and did not have active litigation around affirmative action (S3 Table presents counts of the number of individuals in this sample considered exposed versus unexposed by survey year). In the YRBS, the analytic sample ranged in size from 34,988 (binge drinking) to 36,268 (cigarette smoking) underrepresented minority 11th and 12th graders living in 42 states who were surveyed during 1991–2015. The (weighted) mean age was similar among respondents in states that passed an affirmative action ban versus states that did not (17.1 versus 17.1 years). The weighted percentages of girls versus boys (51.2% versus 51.7%) and 11th versus 12th grade respondents (52.5% versus 49.2%) were also similar in both groups. The states that passed an affirmative action ban at some point during the study period had a higher percentage of Hispanic (versus Black or Native American) respondents (68.7% versus 38.5%).

**Table 1:** Descriptive statistics for YRBS and TUS-CPS study participants.

Characteristic	YRBS, 1991–2015		TUS-CPS, 1992–2015	
	States implementing bans	States not implementing bans	States implementing bans	States not implementing bans
**Age in years, mean (SD)**	17.1 (0.74)	17.1 (0.76)	23.7 (3.3)	23.7 (3.3)
**Sex, *n* (weighted percent)**				
Boys/men	10,103 (48.8)	7,204 (48.3)	14,801 (50.2)	18,938 (48.9)
Girls/women	11,019 (51.2)	7,942 (51.7)	16,139 (49.8)	21,697 (51.1)
**Race/ethnicity, *n* (weighted percent)**				
Black	4,557 (28.3)	8,839 (58.6)	6,214 (21.5)	17,488 (50.3)
Hispanic	16,107 (68.7)	5,965 (38.5)	23,285 (75.3)	19,088 (43.8)
Native American and other	458 (3.0)	343 (2.9)	1,441 (4.1)	4,059 (5.9)
**Grade, *n* (weighted percent)**				
11th	10,394 (52.5)	7,650 (49.2)	N/A	N/A
12th	10,728 (47.6)	7,496 (50.7)	N/A	N/A
***N***	21,122	15,146	30,982	40,729

Descriptive statistics for underrepresented minority 11th and 12th grade students in the 1991–2015 survey waves of the YRBS and 19- to 30-year-old adults in the 1992–2015 survey waves of the TUS-CPS who were likely high school juniors between 1991 and 2015 (i.e., individuals who had attained 16 years of age at any time point between 1990 and 2015). For ease of presentation, the YRBS summary statistics are based on the sample used to estimate the regression models for cigarette smoking (*n* = 36,268). Descriptive statistics using estimation samples from the other regression models were similar. Reported numbers represent raw counts. Percentages and means were computed using sample weights.

N/A, not applicable; TUS-CPS, Tobacco Use Supplement to the Current Population Survey; YRBS, Youth Risk Behavior Survey.

The TUS-CPS sample comprised 71,575 underrepresented minority adults surveyed in 1992–2015. As in the YRBS, the mean age (23.7 versus 23.7 years) and percentage of women (49.8% versus 51.1%) were similar in states that implemented an affirmative action ban compared with those that did not. The percentage of Hispanic (versus Black or Native American) respondents was higher in states implementing bans (75.3% versus 43.8%).

### Difference-in-differences and event study estimates

Table 2 presents the estimated regression coefficients from the difference-in-differences models. Estimates are expressed as absolute percentage point changes. In the YRBS, self-reported cigarette smoking in the past 30 days increased by 3.8 percentage points among underrepresented minority adolescents after exposure to an affirmative action ban (95% CI: 2.0, 5.7; *p <* 0.001). Exposure to a ban was also followed by a 5.9 percentage point increase in self-reported past-30-day alcohol use (95% CI: 0.3, 12.2; *p =* 0.041) and a 3.5 percentage point increase in self-reported past-30-day binge drinking (95% CI: −0.1, 7.2; *p =* 0.058), although the latter estimate was not statistically significant. The *p-*value for the estimate for smoking remained unchanged with adjustment for multiple comparisons. However, the *p*-values for the estimates for both alcohol use outcomes increased (*p =* 0.082 for both). In the TUS-CPS analysis, current smoking increased by 1.8 percentage points in underrepresented minority adults aged 19–30 years after exposure to an affirmative action ban (95% CI: 0.1, 3.6; *p =* 0.037).

**Table 2:** Difference-in-differences estimates for underrepresented minority and non-Hispanic White respondents.

	YRBS			TUS-CPS
	Smoking within past 30 days	Alcohol use within past 30 days	Binge drinking within past 30 days	Current smoking
**Underrepresented minority individuals**				
***Difference-in-differences estimate***	***+3.8% pt***	***+5.9% pt***	***+3.5% pt***	***+1.8% pt***
95% CI	2.0, 5.7	0.3, 12.2	−0.1, 7.2	0.1, 3.6
*p*-Value	*p <* 0.001	*p =* 0.041	*p =* 0.058	*p =* 0.037
*p-*Value adjusting for multiple comparisons	*p <* 0.001	*p =* 0.082	*p =* 0.082	N/A
Weighted prevalence of outcome	19.3%	45.4%	26.3%	13.2%
Estimate relative to sample prevalence	+19.7%	+13.0%	+13.3%	+13.8%
*N*	36,268	35,106	34,988	71,575
**Non-Hispanic White individuals (falsification test)**				
***Difference-in-differences estimate***	***+0.7% pt***	***+1.8% pt***	***+1.7% pt***	***+0.3% pt***
95% CI	−8.0, 9.2	−5.3, 8.9	−5.1, 8.5	−2.1, 2.6
*p-*Value	*p =* 0.880	*p =* 0.612	*p =* 0.622	*p =* 0.825
*p-*Value adjusting for multiple comparisons	*p =* 0.941	*p =* 0.941	*p =* 0.941	N/A
Weighted prevalence of outcome	31.3%	54.0%	38.0%	24.0%
Estimate relative to sample prevalence	+2.1%	+3.3%	+4.5%	+1.3%
*N*	33,082	32,680	32,613	171,095

Difference-in-differences estimates of the association between affirmative action bans and substance use outcomes among underrepresented minority and non-Hispanic White 11th and 12th graders in the YRBS and among 19- to 30-year-old underrepresented minority and non-Hispanic White adults in the TUS-CPS. Estimates are presented as percentage point changes, which are computed by taking the linear probability model regression coefficient and multiplying by 100. The 95% confidence intervals corrected for clustering at the state level are shown. All estimates were adjusted for age, sex, and race/ethnicity (Black, Hispanic, or Native American); state fixed effects; race/ethnic group–year (year of survey in the YRBS, and year when the individual turned 16 years old in the TUS-CPS) fixed effects; and state-specific linear time trends (specific to survey year in the YRBS and the year the individual turned 16 years old in the TUS-CPS). In the YRBS regression models, we additionally adjusted for participant grade (11th versus 12th grade). In the TUS-CPS model, we additionally included fixed effects for the year and month the survey was conducted (i.e., the time when adult outcomes were assessed). We addressed the issue of multiple comparisons in the YRBS analysis, where we have 3 outcome variables. We computed *p-*values for each YRBS outcome, within each racial/ethnic group, using the Sidak–Holm step-down method, which adjusts for the family-wise error rate.

N/A, not applicable; pt, point; TUS-CPS, Tobacco Use Supplement to the Current Population Survey; YRBS, Youth Risk Behavior Survey.

In both the YRBS and TUS-CPS analyses, for all outcomes the estimates for non-Hispanic White individuals were (compared with the estimates for underrepresented minority individuals) smaller in magnitude and not statistically significant—indicating that, as expected, affirmative action bans were not associated with adverse health behaviors in this population.

**Fig 2:**
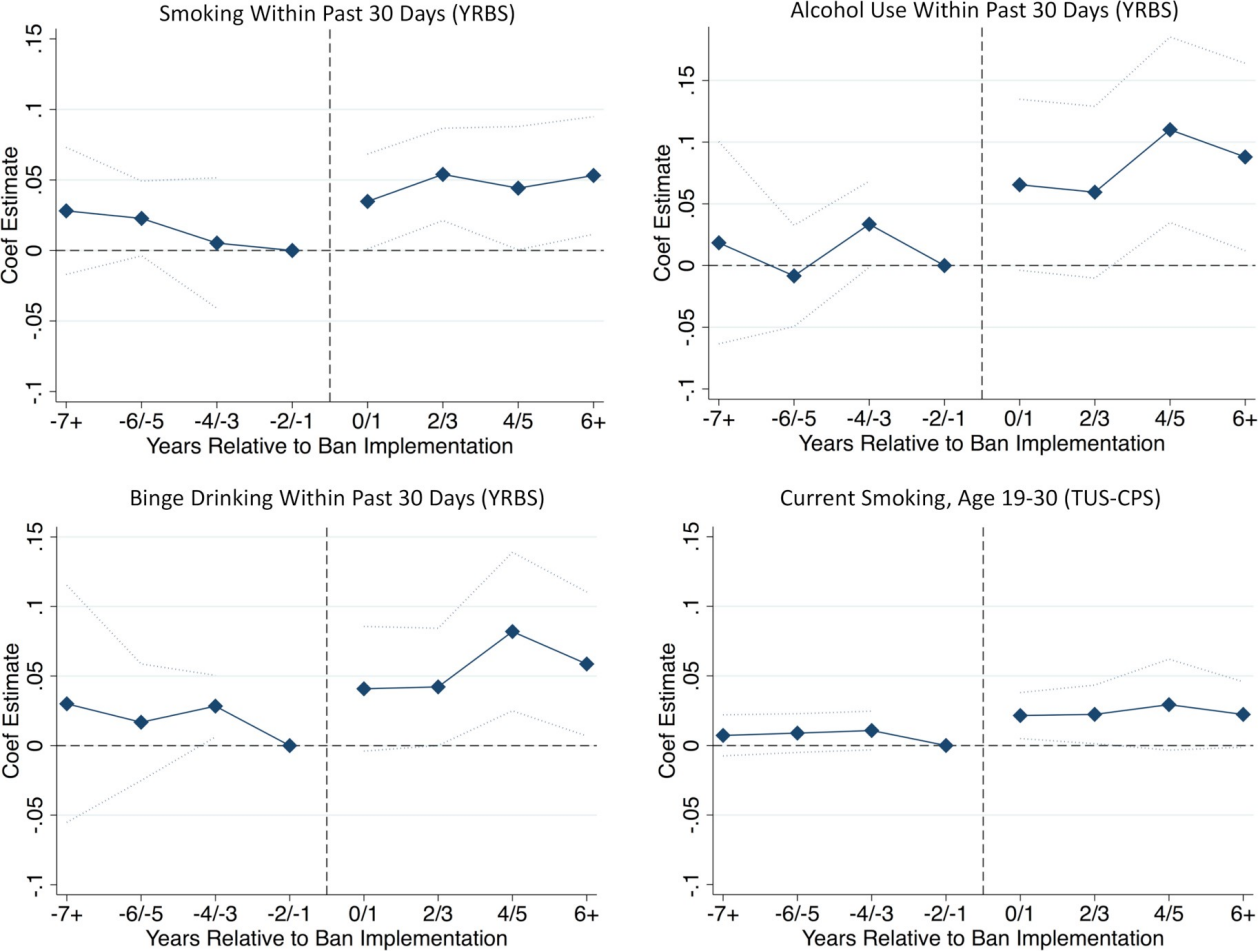
Event study estimates for underrepresented minority respondents. Graphs present event study estimates for underrepresented minority respondents in the YRBS and TUS-CPS. The regression models are identical to those presented in Table 2, except the exposure variable is replaced by a series of binary indicators denoting the timing of interview (or when the individual turned 16 years of age) relative to the policy change. Individuals in states not passing bans were assigned 0 for each of the bins. The 2-year period before ban implementation (event time −2/−1) was denoted as the reference period. The blue diamonds compare the difference in the prevalence of the outcome (“Coef Estimate”), for each point in event time relative to the reference period, between individuals living in states where an affirmative action ban was implemented versus individuals living in states where a ban was not implemented. The dotted blue lines represent 95% confidence intervals, which account for clustering at the state level. There is a confidence interval for the event time period right before ban implementation given that this was denoted as the reference period. Estimates for non-Hispanic White individuals are presented in S2 Fig. TUS-CPS, Tobacco Use Supplement to the Current Population Survey; YRBS, Youth Risk Behavior Survey.

Fig 2 provides a graphical representation of event study estimates for each of the study outcomes. Each point represents the estimated effect of affirmative action bans on the specified outcome for the specified time period (relative to implementation of the ban). The reference group for each estimate includes respondents residing in affected states who were surveyed in the 2-year period just prior to ban implementation, along with respondents residing in unaffected states. For underrepresented minority respondents, the graphical displays show little evidence of preexisting trends in cigarette smoking and alcohol use, while also demonstrating an abrupt increase in the coefficient estimates coinciding with exposure to a ban. The graphical displays also demonstrate that the estimated effects persisted throughout the study period. Consistent with the regression estimates, there was little evidence of a discrete change in any of the outcomes among non-Hispanic White respondents (S2 Fig). The magnitudes of the estimates were not consistently larger for boys/men versus girls/women, or for Black versus Hispanic respondents (S4 Table).

### Sensitivity analyses

Estimates were robust to inclusion of state policy and economic variables, accounting for potential spillover effects in neighboring states, exposure reclassification (from exposed to unexposed) for adolescents living in Texas in 2003, and restriction of the analytic sample to respondents in states that passed an affirmative action ban at some point during the study period (S5 Table). Using the TUS-CPS data, we tested for an effect of a negative control exposure and found that underrepresented minority adults exposed to an affirmative action ban after high school did not show an increase in cigarette smoking during adulthood (S6 Table). Finally, using the CPS-ASEC data, we found no evidence of selective migration or changes in high school dropout rates subsequent to affirmative action ban implementation (S7 Table).

## Discussion

In this nationally representative study of US adolescents, we found that rates of cigarette smoking among underrepresented minority adolescents increased after exposure to affirmative action bans. Concern about these acute and contemporaneous adverse effects was corroborated and further magnified by our finding, in a separate dataset, that the apparent effects of affirmative action bans on smoking persisted into young adulthood. We also found evidence of apparent increases in alcohol use and binge drinking after exposure to affirmative action bans, though the estimates did not remain statistically significant at conventional thresholds after adjustment for multiple comparisons. As expected, we found no evidence of changes in health risk behaviors after affirmative action bans in our falsification sample of non-Hispanic White individuals, and results were robust to several other specification checks designed to probe the key causal identification assumptions of our difference-in-differences model.

Our findings have 2 important implications for policy and practice. First, the results suggest that health behaviors respond to changes in socioeconomic opportunities driven by changes in social policy. Our findings provide rare evidence supporting new hypotheses about the importance of economic opportunity for population health [5,8,9]. The findings also complement a growing literature documenting strong relationships between socioeconomic factors and health and human capital investments among adolescents and young adults [5,56–60].

Second, our study has important implications for ongoing debates over race-based affirmative action policies. The impacts of race-based affirmative action programs on educational and economic opportunities for underrepresented minorities are well known [16,18,19,27,61]. However, their effects on population health have heretofore not been well studied [62]. Our study suggests that ongoing efforts to ban affirmative action programs in college admissions [13–15] may have significant unanticipated adverse effects on health risk behaviors and health status within underrepresented minority populations. In doing so, they may exacerbate short- and long-run disparities in health outcomes. This possibility is particularly noteworthy given that the negative consequences of health risk behaviors are amplified for individuals of underrepresented minority groups, who tend to experience greater adverse health and socioeconomic consequences than White individuals for the same behaviors [63–65].

The interpretation of our findings is subject to several limitations. First, the outcomes were all self-reported. However, the YRBS design includes detailed safeguards to protect the confidentiality of study participants [32], and the documented accuracy of self-reported substance use in the YRBS reflects favorably on these safeguards [66]. Second, it is possible that there remain unmeasured time-varying state-level factors correlated with the outcomes and with the timing of implementation of affirmative action bans in ways that could potentially bias our estimates. While unmeasured confounding cannot be definitively ruled out, our findings were supported by an event study specification, were robust to changes in specification, and were replicated in 2 distinct datasets. Moreover, the specificity of our estimates was supported by our use of a falsification test and negative controls.

Third, we examined affirmative action bans that occurred up to 20 years in the past, when the epidemiology of health risk behaviors among adolescents may have differed from that of the present. It is possible that contemporaneous and future affirmative action policy changes may manifest in a different patterning of health risk behaviors. Fourth, we were unable to analyze the mediating pathways linking affirmative action bans to health risk behaviors. Neither dataset measured perceptions of economic opportunities, racism, or fairness or contained information on other mediators such as stress, anxiety, or depression during the study period. (Some relevant variables were collected in the YRBS but only after the largest states had already implemented their affirmative action bans.) Elucidating the mechanisms underlying our findings will remain an important topic for future research.

### Implications

State policies banning race-based affirmative action in college admissions appear to have led to increases in health risk behaviors among underrepresented minority adolescents during the time period studied. The adverse impacts persisted into adulthood and were specific to underrepresented minorities. The potentially important population health consequences of social and economic policy changes should be recognized in ongoing policy debates. Policymakers, public health practitioners, and clinicians should consider these health consequences as part of the overall evaluation of the benefits and costs of social and economic policies.

## Supporting information

S1 Appendix Materials and methods. (DOCX)

S1 Fig Trends in media coverage of affirmative action bans, relative to their implementation. (DOCX)

S2 Fig Event study estimates for non-Hispanic White respondents. (DOCX)

S1 STROBE checklist (DOCX)

S1 Table States implementing college admission affirmative action bans, 1991–2015. (DOCX)

S2 Table YRBS coverage, by state and by year. (DOCX)

S3 Table Exposed and unexposed observations by survey year and outcome (YRBS). (DOCX)

S4 Table Difference-in-differences estimates, by race and sex. (DOCX)

S5 Table Sensitivity analyses for difference-in-differences estimates. (DOCX)

S6 Table Falsification test for the TUS-CPS. (DOCX)

S7 Table Analysis of nonrandom selection due to high school dropout and migration. (DOCX)

S8 Table Analyses with and without sampling weights. (DOCX)

## Abbreviations

CPS-ASEC: Annual Social and Economic Supplement of the Current Population Survey
TUS-CPS: Tobacco Use Supplement to the Current Population Survey
YRBS: Youth Risk Behavior Survey

## References

[1] Bor J, Cohen GH, Galea S. Population health in an era of rising income inequality; USA, 1980–2015. Lancet. 2017;389:1475–90. 10.1016/S0140-6736(17)30571-8 28402829

[2] Chetty R, Stepner M, Abraham S, Lin S, Scuderi B, Turner N, et al The association between income and life expectancy in the United States, 2001–2014. JAMA. 2016;315(16):1750–66. 10.1001/jama.2016.4226 27063997 PMC4866586

[3] Ezzati M, Friedman A, Kulkarni S, Murray C. The reversal of fortunes: trends in county mortality and cross-county mortality disparities in the United States. PLoS Med. 2008;5(4):e66 10.1371/journal.pmed.0050066 18433290 PMC2323303

[4] Marmot MG, Bell R. Action on health disparities in the United States: commission on social determinants of health. JAMA. 2009;301(11):1169–71. 10.1001/jama.2009.363 19293419

[5] Ananat EO, Gassman-Pines A, Francis DV, Gibson-Davis CM. Linking job loss, inequality, mental health, and education. Science. 2017;356(6343):1127–8. 10.1126/science.aam5347 28619903

[6] Case A, Deaton A. Rising morbidity and mortality in midlife among white non-Hispanic Americans in the 21st century. Proc Natl Acad Sci USA. 2015;112(49):15078–83. 10.1073/pnas.1518393112 26575631 PMC4679063

[7] Ludwig J, Sanbonmatsu L, Gennetian L, Adam E, Duncan GJ, Katz LF, et al Neighborhoods, obesity, and diabetes—a randomized social experiment. N Engl J Med. 2011;365:1509–19. 10.1056/NEJMsa1103216 22010917 PMC3410541

[8] Venkataramani AS, Chatterjee P, Kawachi I, Tsai AC. Economic opportunity, health behaviors, and mortality in the United States. Am J Public Health. 2016;106(3):478–84. 10.2105/AJPH.2015.302941 26691108 PMC4758869

[9] Kearney MS, Levine PB. Income inequality and early nonmarital childbearing. J Hum Resour. 2014;49(1):1–31.

[10] Venkataramani AS, Shah SJ, O’Brien R, Kawachi I, Tsai AC. Health consequences of the US Deferred Action for Childhood Arrivals program. Lancet Pub Health. 2017;2:e175–81. 29253449 10.1016/S2468-2667(17)30047-6 PMC6378686

[11] Smedley BD, Stith AY, Nelson AR, editors. Unequal treatment: confronting racial and ethnic disparities in health care. Washington (DC): National Academies Press; 2003. 25032386

[12] Chetty R, Hendren N, Jones MR, Porter SR. Race and economic opportunity in the United States: an intergenerational perspective. NBER Working Paper No. 24441. Cambridge (MA): National Bureau of Economic Research; 2018.

[13] Savage C. Justice Dept. to take on affirmative action in college admissions. The New York Times. 2017 Aug 1 [cited 2019 May 14]. Available from: https://www.nytimes.com/2017/08/01/us/politics/trump-affirmative-action-universities.html.

[14] Hartocollis A. Does Harvard admissions discriminate? The lawsuit on affirmative action, explained. The New York Times. 2018 Oct 15 [cited 2019 May 14]. Available from: https://www.nytimes.com/2018/10/15/us/harvard-affirmative-action-asian-americans.html.

[15] Jaschik S. Affirmative action fight shifts to UNC. Inside Higher Ed. 2019 Jan 22 [cited 2019 May 14]. Available from: https://www.insidehighered.com/admissions/article/2019/01/22/legal-fight-over-affirmative-action-shifts-unc-chapel-hill.

[16] Backes B. Do affirmative action bans lower minority college enrollment and attainment? Evidence from statewide bans. J Hum Resour. 2012;47(2):435–55.

[17] Cortes KE. Do bans on affirmative action hurt minority students? Evidence from the Texas top 10% plan. Econ Educ Rev. 2010;29(6):1110–24.

[18] Hinrichs P. The effects of affirmative action bans on college enrollment, education, and demographic consequences of universities. Rev Econ Stat. 2012;94(3):712–22.

[19] Hinrichs P. Affirmative action bans and college graduation rates. Econ Educ Rev. 2014;42(43–52):43–52.

[20] Dickson LM. Does ending affirmative action in college admissions lower the percent of minority students applying to college? Econ Educ Rev. 2006;25:109–19.

[21] Leventhal AM, Cho J, Andrabi N, Barrington-Trimis J. Association of reported concern about increasing societal discrimination with adverse behavioral health outcomes in late adolescence. JAMA Pediatr. 2018;172(10):924–33. 10.1001/jamapediatrics.2018.2022 30128537 PMC6233761

[22] Bailey ZD, Krieger N, Agenor M, Graves J, Linos N, Bassett MT. Structural racism and health inequalities in the USA: evidence and interventions. Lancet. 2017;389(10077):P1453–63. 10.1016/S0140-6736(17)30569-X 28402827

[23] Bor J, Venkataramani AS, Williams DR, Tsai AC. Police killings and their spillover effects on the mental health of black Americans: a population-based, quasi-experimental study. Lancet. 2018;392:302–10. 10.1016/S0140-6736(18)31130-9 29937193 PMC6376989

[24] Fryer RG, Loury GC, Yuret T. An economic analysis of color-blind affirmative action. J Law Econ Organ. 2008;2:319–55.

[25] King M, Jennings J, Fletcher JM. Medical adaptation to academic pressure: schooling, stimulant use, and socioeconomic status. Am Sociol Rev. 2014;79(6):1039–66.

[26] Dudovitz RN, Chung PJ, Reber S, Kennedy D, Tucker JS, Shoptaw S, et al Assessment of exposure to high-performing schools and risk of adolescent substance use: a natural experiment. JAMA Pediatr. 2018;172(12):1135–44. 10.1001/jamapediatrics.2018.3074 30383092 PMC6350909

[27] Bodoh-Creed AL, Hickman BR. Pre-college human capital investment and affirmative action: a structural policy analysis of US college admissions. Working Paper. Berkeley (CA): Haas School of Business; 2018 [cited 2019 May 24]. Available from: http://faculty.haas.berkeley.edu/acreed/AA_Empirical.pdf.

[28] Bodoh-Creed AL, Hickman BR. College assignment as a large contest. J Econ Theory. 2018;175:88–126.

[29] Ganz ML. The relationship between external threats and smoking in central Harlem. Am J Public Health. 2000;90:367–71. 10.2105/ajph.90.3.367 10705853 PMC1446181

[30] Godfrey EB, Burson E, Santos CE. For better or worse? System-justifying beliefs in six-grade predict trajectories of self-esteem and behavior across early adolescence. Child Dev. 2019;90(1): 180–195. 10.1111/cdev.12854 28631266

[31] McDade TW, Chyu L, Duncan GJ, Hoyt LT, Doane LD, Adam EK. Adolescents’ expectations for the future predict health behaviors in early adulthood. Soc Sci Med. 2011;(73):3. 10.1016/j.socscimed.2011.06.005 PMC3148854 21764487

[32] Brener ND, Kann L, Shanklin S, Kinchen S, Eaton D, K, Hawkins J. Methdology of the Youth Risk Behavior Surveillance System—2013. MMWR Recomm Rep. 2013;62(RR-1):1–20. 23446553

[33] Wu F. Neither black nor white: Asian Americans and affirmative action. Boston Coll Third World Law J. 1995;15:225–84.

[34] Chung Allred N. Asian Americans and affirmative action: from yellow peril to model minority and back again. Asian Am Law J. 2007;14:57–84.

[35] Museus SA. Asian American students in higher education. New York: Routledge; 2014.

[36] Dupere V, Leventhal T, Dion E, Crosnoe R, Archambault I, Janosz M. Stressors and turning points in high school dropbout: a stress process, life course framework. Rev Educ Res. 2015;85(4):591–629.

[37] Leonard NR, Gwadz MV, Ritchie A, Linick J, Cleland CM, Elliot L, et al A multi-method exploratory study of stress, coping, and substance use among high school youth in private schools. Front Psychol. 2015;6:1028 10.3389/fpsyg.2015.01028 26257685 PMC4511824

[38] Flood S, King M, Ruggles S, Warren JR. Integrated public use microdata series, Current Population Survey. Version 5.0. Minneapolis: University of Minnesota; 2017.

[39] Angrist JD, Pischke J-S. Mastering metrics: the path from cause to effect. Princeton: Princeton University Press; 2015.

[40] Card D, Krueger AB. Minimum wages and employment: a case study of the fast-food industry in New Jersey and Pennsylvania. Am Econ Rev. 1994;84(4):772–793.

[41] Greene W. The behavior of the maximum likelihood estimator of limited dependent variable models in the presence of fixed effects. Econ J. 2004;7(1):98–119.

[42] Dornbusch SM, Mont-Reynaud R, Ritter PL, Chen Z, Steinberg L. Stressful events and their correlates among adolescents of diverse backgrounds In: Colten ME, Gore S, editors. Adolescent stress: causes and consequences. New York: Gruyter; 1991.

[43] Mahalik JR, Levine Coley R, McPherran Lombardi C, Doyle Lynch A, Markowitz A, Jaffee S. Changes in health risk behaviors for males and females from early adolescence through early adulthood. Health Psychol. 2013;32(6):685–94. 10.1037/a0031658 23477574

[44] Silberg J, Pickles A, Rutter M, Hewitt J, Simonoff E, Maes H, et al The influence of genetic factors and life stress on depression among adolescent girls. Arch Gen Psychiatry. 1999;56(3):225–32. 10078499 10.1001/archpsyc.56.3.225

[45] Goodman-Bacon A. Difference-in-differences with variation in treatment timing. NBER Working Paper No. 25018. Cambridge (MA): National Bureau of Economic Research; 2018.

[46] Prasad V, Jena AB. Prespecified falsification end points: can they validate true observational associations? JAMA. 2013;309(3):241–2. 10.1001/jama.2012.96867 23321761

[47] Lipsitch M, Tchetgen Tchetgen E, Cohen T. Negative controls: a tool for detecting confounding and bias in observational studies. Epidemiology. 2010;21(3):383–8. 10.1097/EDE.0b013e3181d61eeb 20335814 PMC3053408

[48] Bertrand M, Dulfo E, Mullainathan S. How much should we trust differences-in-differences estimates? Q J Econ. 2004;119(1):249–75.

[49] Holm S. A simple sequentially rejective multiple test procedure. Scand J Stat. 1979;6(2):65–70.

[50] Sidak Z. Rectangular confidence regions for the means of multivariate normal distributions. J Am Stat Assoc. 1967;62:626–33.

[51] Anderson ML. Multiple inference and gender differences in the effects of early intervention: a reevaluation of the Abecedarian, Perry Preschool, and Early Training projects. J Am Stat Assoc. 2008;103(484):1481–95.

[52] Solon G, Haider SJ, Wooldridge JM. What are we weighting for? J Hum Resour. 2015;50(2):301–16.

[53] Carpenter C, Cook PJ. Cigarette taxes and youth smoking: new evidence from national, state, and local Youth Risk Behavior Surveys. J Health Econ. 2008;27(2):287–99. 10.1016/j.jhealeco.2007.05.008 18242745

[54] Xu X, Chaloupka FJ. The effects of prices on alcohol use and its consequences. Alcohol Res Health. 2011;34(2):236–45. 22330223 PMC3860576

[55] Blume GH, Long MC. Changes in levels of affirmative action in college admissions in response to statewide bans and judicial rulings. Educ Eval Policy Anal. 2014;36(2):228–52.

[56] Cascio E, Narayan A. Who needs a fracking education? The educational response to low-skill biased technological change. NBER Working Paper No. 21359. Cambridge (MA): National Bureau of Economic Research; 2015.

[57] Kuka E, Shenhav Na, Shih K. Do human capital decisions respond to the returns to education? Evidence from DACA. NBER Working Paper No. 24315. Cambridge (MA): National Bureau of Economic Research; 2018.

[58] Cowan BW. Forward-thinking teens: the effects of college costs on adolescent risky behavior. Econ Educ Rev. 2011;30(5):813–25. 10.1016/j.econedurev.2011.04.006 21886942 PMC3163495

[59] Hao Z, Cowan BW. The effects of graduation requirements on risky health behaviors of high school students. Am J Health Econ. 2017;5(1):97–125.

[60] Raifman J, Moscoe E, Austin S, McConnell M. Difference-in-differences analysis of the association between state same-sex marriage policies and adolescent suicide attempts. JAMA Pediatr. 2017;171(4):350–6. 10.1001/jamapediatrics.2016.4529 28241285 PMC5848493

[61] Arcidiacono P, Lovenheim M. Affirmative action and the quality-fit trade-off. J Econ Lit. 2016;54(1):3–51.

[62] Williams DR, Jackson PB. Social sources of racial disparities in health. Health Aff (Millwood). 2005;24(2):325–34. 15757915 10.1377/hlthaff.24.2.325

[63] Witbrodt J, Mulia N, Zemore S, Kerr W. Racial/ethnic disparities in alcohol-related problems: differences by gender and level of heavy drinking. Alcohol Clin Exp Res. 2014;38(6):1662–70. 10.1111/acer.12398 24730475 PMC4047188

[64] Gadgeel S, Kalemkerian G. Racial differences in lung cancer. Cancer Metastasis Rev. 2003;22(1):39–46. 12716035 10.1023/a:1022207917249

[65] Kakade M, Duarte CS, Liu X, Fuller CJ, Drucker E, Hoven CW, et al Adolescent substance use and other illegal behaviors and racial disparities in criminal justice system involvement: findings from a US national survey. Am J Public Health. 2012;102(7):1307–10. 10.2105/AJPH.2012.300699 22594721 PMC3477985

[66] Rosenbum JE. Truth or consequences: the intertemporal consistency of adolescent self-report on the Youth Risk Behavior Survey. Am J Epidemiol. 2009;169(11):1388–97. 10.1093/aje/kwp049 19363096 PMC2727247

