# Supplementary material for: College affirmative action bans and smoking and alcohol use among underrepresented minority adolescents in the United States: A difference-in-differences study
Source: PLoS Med. 2019 Jun 18;16(6):e1002821. doi: 10.1371/journal.pmed.1002821 (PMC6581254; doi:10.1371/journal.pmed.1002821)
Supplement: S1 Appendix — (DOCX) [file pmed.1002821.s001.docx]

**S1 Appendix. Materials and Methods**

*U.S. National Youth Risk Behavior Survey*

The U.S. National Youth Risk Behavior Survey (YRBS) is a nationally representative, repeated cross-sectional survey of 9th-12th graders in public and private schools fielded by the U.S. Centers for Disease Control and Prevention (CDC) as part of the national Youth Risk Behavior Surveillance System (YRBSS). Surveys have been conducted biennially since 1991, typically in the spring. All data were publicly available and de-identified, so we did not seek institutional review board approval to conduct the study.

We chose the YRBS over other comparable datasets because the YRBS provides a national sample of 11^th^ and 12^th^ graders, the adolescent subgroup for whom college decisions are most salient; its surveys coincided with most of the affirmative action bans that have been implemented in U.S. history; and it offers health behavior data for adolescents surveyed before and after affirmative action bans in 7 of the 9 implementing states, including continuous data collection before and after affirmative action bans for Texas and California, the largest states implementing bans (S2 Table). (The two remaining states, Nebraska and New Hampshire, were both surveyed during a single wave, prior to implementation of bans).

Other datasets did not provide this degree of coverage and were therefore not appropriate for our analysis. For example, the National Longitudinal Study of Adolescent to Adult Health does not provide survey coverage of high school cohorts around the time of affirmative action ban implementation; the Monitoring the Future survey does not survey 11^th^ graders; and the National Longitudinal Surveys of Youth features a much smaller sample size in comparison. The CDC YRBSS also supports surveys that are representative at the level of the state. These data, however, were also not appropriate for this analysis given that they did not provide continuous coverage for either Texas or California and only provided continuous coverage for 2 of the 9 states implementing affirmative action bans during the study period.

YRBS Outcomes

We focused on cigarette smoking and alcohol use as the primary outcomes of interest for several reasons. First, these outcomes are of intrinsic importance to adolescent health. Second, the survey questions corresponding to these outcomes had a short duration of recall. Since the YRBS is generally conducted in the Spring semester, longer recall windows will potentially capture substance use in the prior year. Third, these outcomes were consistently available across all waves of the YRBS.

The specific survey items were as follows:

1. Self-reported cigarette use: “During the past 30 days, on how many days did you smoke cigarettes?”
2. Self-reported alcohol use: “During the last 30 days, on how many days did you have at least one drink of alcohol?”
3. Self-reported binge drinking: “During the past 30 days, on how many days did you have 5 or more drinks of alcohol in a row, that is, within a couple of hours?”

For each of these questions, the response options were limited to: 0 days; 1 or 2 days; 3 to 5 days; 6 to 9 days; 10-19 days; 20 to 29 days; and 30 days. Individuals reporting at least 1 day or more were coded as having engaged in that behavior.

We did not consider other outcomes in the YRBS because they failed to meet one or more of the criteria described above. Illicit and recreational drug use were elicited with 1 year or lifetime recall windows. Questions on depression and anxiety were not fielded in the YRBS until 1999, well after Texas and California had implemented their respective affirmative action bans. Sexual risk behaviors were elicited with recall windows that potentially extended to the prior year.

Of the 39,992 underrepresented minority 11^th^ and 12^th^ grade participants (the sample which excludes individuals residing in four states - Alabama, Georgia, Louisiana, and Mississippi - where there had been ongoing (i.e., multi-year) litigation around affirmative action during the study period, without actual implementation of bans), 1,602 (4.0%) did not have recorded information on state of residence. These observations were dropped. Thereafter, we also dropped the 93 observations for which there was no information recorded for sex and/or age. Of the remaining 38,297 observations, only 2,029 (5.3%) had missing observations for smoking in the last 30 days prior to survey; 3,191 (8.3%) had missing data for alcohol use in the last 30 days; and 3,309 (8.6%) had missing data for binge drinking in the last 30 days. Importantly, for each outcome, the probability of missingness was unrelated to the passage and implementation of affirmative action bans. In difference-in-differences models of the same specification as presented in the main text using missingness as the primary outcome, the coefficient on affirmative action bans was neither substantively or statistically significant (smoking: b=-0.017 [95% CI: -0.044, 0.010], p=0.212; alcohol: b=-0.017 [95% CI: -0.038, 0.003], p=0.099; binge drinking: b=-0.018 [95% CI: -0.039, 0.003], p=0.096).

*Current Population Survey Tobacco Use Supplements*

The Current Population Survey Tobacco Use Supplements (TUS-CPS) is a nationally- and state-representative repeated cross-sectional survey of the U.S. general population, with individuals aged 18 years and older consistently surveyed in all waves. The data have been collected across eight survey cycles from 1992 to 2015. Each cycle includes up to three surveys. In total, the 16 surveys were conducted in 1992-1993, 1995-1996, 1998-1999, 2000, 2001-2002, 2003, 2006-2007, 2010-2011, and 2014-2015.

TUS-CPS Outcome

Individuals who reported smoking at least 100 cigarettes in their lifetime were asked the following question about current smoking:

“(Do/Does) (you/name) now smoke cigarettes every day, some days, or not at all?

Response options were: “every day, “some days,” or “not at all.” Individuals answering either “every day” or “some days” were considered current smokers.

We focused on the same cohorts as in the YRBS, i.e., underrepresented racial and ethnic minorities who were likely to be juniors and seniors in high school between 1991-2015. To do so, we focused on individuals who turned 16 years of age (the time most individuals start 11^th^ grade) during or after 1990 (the sample covers the 1974-1996 birth cohorts). We further restricted the TUS-CPS sample to those aged 19-30 years at the time of the survey, so as to focus on the population of young adults who had (likely) already exited high school. Of the 71,711 underrepresented minority respondents meeting our age and survey year inclusion criteria, only 136 (0.21%) had missing data for the primary outcome.

Of note, between 1992 and 2007, 15-17 year-olds were surveyed in the TUS-CPS. However, we did not examine current smoking outcomes in this group because the question was administered only to those who reported they had smoked at least 100 cigarettes in their lifetime. This questionnaire skip pattern makes it impossible to reconcile the TUS-CPS adolescent smoking estimates with the YRBS smoking estimates.

*Statistical Analysis*

Difference-in-Differences Regression Models Fitted to YRBS Data

Difference-in-differences estimates were obtained by fitting the following linear probability models:

$$Y_{ijt}= \beta_{0}+ \beta_{1}{AABan}_{jt}+ \boldsymbol{\beta X}_{\boldsymbol{ijt}}+ \mu_{j}+ \theta_{t}+ \mu_{j}t+ \varepsilon_{ijt}$$

where *i* indexes the individual study participant, *j* the state of residence, and *t* the survey year. ***Y_ijt_*** denotes the outcome of interest. The key covariate of interest is ***AABan_jt_***, a binary indicator = 1 if state *j* had an active affirmative action ban in place in year *t* and zero otherwise. The difference-in-differences estimate is denoted by *β_1_*, which recovers the average change in the health behavior of interest (before vs. after the ban) among under-represented minority adolescents residing in states implementing bans versus those that did not.

The regression models included binary indicators for age categories, sex, and race/ethnicity (Black, Hispanic, Native American), denoted collectively by ***X_ijt_***. State fixed effects (denoted by *μ_i_*) captured time-invariant differences in socioeconomic, cultural, and political characteristics of states that could be correlated with affirmative action policy adoption and with the outcomes. Nationwide racial/ethnic group-specific trends in the outcomes were captured with a series of survey year-racial/ethnic group fixed effects (denoted by *θ_t_*). Any differential trends in the outcomes over the study period that may differ across states were captured by state-specific linear time trends (denoted by *μ_i_t*). We estimated the model using least squares, given well known biases in limited dependent variable models with panel data (Greene W. Econ J 2004;7:98-119).

Difference-in-Differences Regression Models Fitted to TUS-CPS Data

The difference-in-differences regression model fitted to the TUS-CPS data was largely similar to the model fitted to the YRBS data. The primary exposure of interest was whether an affirmative action ban was in place in the respondent’s state of current residence during the year the respondent turned 16 years old (which was estimated using survey year and current age). This variable captured exposure to affirmative action bans during the high school years. Some non-differential measurement error is to be expected given that the respondent’s state of residence at the time of the TUS-CPS and the respondent’s state of residence during high school might not coincide. However, any bias resulting from misclassification of state of exposure is likely to be small. In an ancillary analysis of data from the 2001-2016 American Community Surveys (ACS), we found that over 75% of 19-30-year-olds reported living in the same state at the time of the survey as the state in which they were born. There was also no substantive difference in the likelihood of migrating from the state of birth for those exposed versus not exposed to affirmative action bans during high school.

Importantly, the analog to the survey year fixed effects in the YRBS in the TUS-CPS are fixed effects for the year the individual was 16 years old. State-specific linear time trends were defined specific to this time point. In addition, we also include TUS-CPS survey year and month fixed effects, which capture the timing of when adult smoking outcomes are assessed.

Event Study Regression Models

The event study estimates were obtained from the following regression model:

$$Y_{ijt}= \alpha_{0}+\sum_{p=-4}^{-2} \alpha_{p}\left( {BanPeriod}_{jp} \right) + {\sum_{p=0}^{3} \alpha_{p}\left( {BanPeriod}_{jp} \right)\boldsymbol{+\beta X}}_{\boldsymbol{ijt}}+ \mu_{j}+ \theta_{t}+\mu_{j}t+ \varepsilon_{ijt}$$

The subscript *p* refers to the event period, which are groupings of event time into two-year windows for the event period 8 years before and 7 years after the passage of affirmative action bans in state *j*. For example, period -4 refers to 7 or more years before policy implementation and period = 0 refers to the year of and year after ban implementation. The variable *BanPeriod_jp_* denotes a series of binary indicators = 1 if an affirmative action ban was in place in state *j* in event-time period *p.* This model estimates the relative difference in outcomes for leads and lags of ban implementation relative to a reference year (period = -1, or the two years just before the ban was enacted) and relative to all states that did not pass affirmative action bans (i.e., for whom *BanPeriod_jp_* is equal to zero for all event periods). These relative differences are captured by the coefficients *α_p_*. We used two-year windows given that neither the YRBS nor the TUS-CPS was conducted annually.

Sampling Weights

We did not apply YRBS or CPS-TUS sampling weights in estimating the regression models for several reasons. First, the use of sampling weights may greatly increase standard errors in analyses where individual-level error terms are clustered within a larger group (as they are in the present application, where students are clustered within states, and the state is the level of policy variation). Second, the surveys were not purposively sampled by the outcome of interest, and we did not *a priori* expect a large degree of heterogeneity in the estimated effects. These happen to be two situations in which weighting is appropriate for models seeking to estimate causal effects (Solon G, Haider SJ, Wooldridge JM. J Hum Resources 2015;50:301-16). Third, in the case of the YRBS, sampling was based on sex, race, and grade, but we flexibly adjusted for these variables in our regression models.

The above discussion implies that use of the sampling weights in this particular application would yield inefficient estimates relative to unweighted models. Consistent with econometric theory and our *a priori* expectations, we found that the weighted and unweighted models yielded substantively similar point estimates but that the weighted estimates were less precise (S8 Table).

Multiple Comparisons

We addressed multiple comparisons in the YRBS analyses given that there were 3 outcome variables of interest. Specifically, we used the Sidak-Holm step-down method to compute *p­-*values for each model, which adjusts for the family-wise error rate and therefore is less likely to under-reject the null hypothesis compared with a standard Bonferroni correction (Holm S, Scand J Statist 1979;6:65-70. Sidak Z, J Am Stat Assoc 1967;62:626-33). We conducted this procedure separately for the underrepresented minority student sample and the non-Hispanic White student sample, given our *ex ante* hypothesis that these groups would be differentially affected by affirmative action bans (Anderson ML, J Am Stat Assoc 2008;103:1481-95).

*Prespecified Analysis Plan*

No prospective protocol was published or registered for this observational study. However, we followed a clear analysis plan, as described in the methods section, and did not deviate from this plan.

- The inclusion/exclusion criteria for the study were established at the outset and were not changed. We used data from the 1991-2015 YRBS, a nationally representative, repeated cross-sectional survey of 9th-12th graders in public and private schools fielded by the CDC as part of the national YRBSS. We restricted the sample to 11th and 12th grade students to focus on the distinct developmental stage of late high school, when decisions about college and future careers are particularly salient.
- The statistical analyses were determined at the outset and were not changed (although we later added sensitivity analyses to explore the robustness of our findings to alternative specifications). The primary analysis focused on estimating difference-in-differences regression models to compare changes in outcomes before and after exposure to affirmative action bans among under-represented minority respondents residing in affected states versus those residing in non-affected states.
- All subgroup analyses were pre-specified and theoretically motivated. Estimation focused on under-represented minority students, defined as those who self-reported their race as “Black” or who self-reported their ethnicity as “Hispanic” or “Native American.” Given the role of affirmative action in remediating historically and structurally ingrained racial inequalities, we hypothesized that non-Hispanic White students would be differentially affected by affirmative action bans. We did not include data on Asian-American and Pacific Islander students because their sample sizes were too small for robust inference.
- In addition to estimating the regression models for the pooled sample of under-represented minorities, we also estimated models stratifying by sex and race/ethnicity (Black vs. Hispanic), given potential differences in responses to stressful life events across these groups.
- Our outcomes were determined at the outset: any self-reported cigarette smoking, alcohol use, and/or binge drinking in the 30 days prior to survey.
- In the original analysis plan, we did not specify that the CPS-TUS data would be used to examine for potential persistence of smoking impacts into adulthood. After conducting the primary analysis using the YRBS data, however, we realized the CPS-TUS data could be used to (a) corroborate potential persistence in smoking behavior and (b) verify our findings in an alternate sample that is representative at the state level. For these reasons, we added it as a supplementary analysis.
- In the original analysis plan, we did not explicitly account for multiple comparisons given that all outcome analyses were theoretically motivated. In response to reviewer comments, we additionally accounted using the Sidak-Holm step-down method to compute p-values for each outcome that adjust for the family-wise error rate. We conducted this procedure separately for the under-represented minority student sample and the non-Hispanic White student sample, given our *ex ante* hypothesis that these groups would be differentially affected by affirmative action bans.
