# Supplementary material for: College affirmative action bans and smoking and alcohol use among underrepresented minority adolescents in the United States: A difference-in-differences study
Source: PLoS Med. 2019 Jun 18;16(6):e1002821. doi: 10.1371/journal.pmed.1002821 (PMC6581254; doi:10.1371/journal.pmed.1002821)
Supplement: S1 Fig — (DOCX) [file pmed.1002821.s002.docx]

**S1 Figure.** Trends in Media Coverage of Affirmative Action Bans, Relative to Their Implementation

**
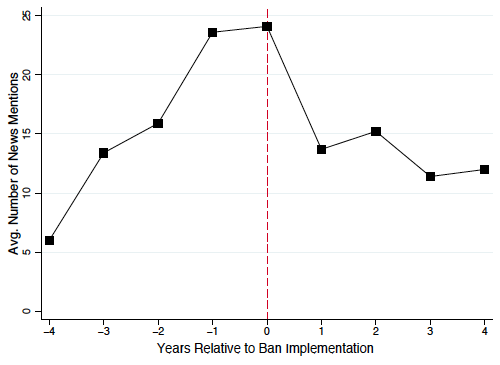
**

**Notes:** The graph represents the average number of news mentions per state-year in the four years before and after policy implementation, in the 9 states that implemented affirmative action bans during the study period. The sample includes the 9 states that passed affirmative action bans during the 1991-2015 time period covered by our study. For each state, we identified news mentions in the LexisNexis database over the period 1990-2015 using the following search terms: (affirmative action) AND (ban) AND (college OR university) AND (admission) AND ([state name]). The year in which each state passed a ban is designated as event-time point zero and the time periods before and after passage are designated as negative and positive integers
