# Supplementary material for: College affirmative action bans and smoking and alcohol use among underrepresented minority adolescents in the United States: A difference-in-differences study
Source: PLoS Med. 2019 Jun 18;16(6):e1002821. doi: 10.1371/journal.pmed.1002821 (PMC6581254; doi:10.1371/journal.pmed.1002821)
Supplement: S2 Fig — (DOCX) [file pmed.1002821.s003.docx]

**S2 Figure.** Event Study Estimates for Non-Hispanic White Respondents

**Notes**: Graphs present event study estimates for non-Hispanic White respondents in the YRBS and TUS-CPS similar to those presented in **Figure 2** for under-represented minority respondents. The regression models are identical to those presented in **Table 2** of the main text, except the exposure variable is replaced by a series of binary indicators denoting the timing of interview (or when the individual was 16 years of age) relative to the policy change. Individuals in states not passing bans were assigned zero for each of the bins. The maroon squares compare the difference in the prevalence of the outcome (“Coef Estimate”), for each point in event time relative to the reference period, between individuals living in states where an affirmative action ban was implemented versus individuals living in states where a ban was not implemented. The dotted blue lines represent 95% confidence intervals, which account for clustering at the state level. The two-year period before ban implementation (event time -2/-1) was denoted as the reference period.
