# Supplementary material for: College affirmative action bans and smoking and alcohol use among underrepresented minority adolescents in the United States: A difference-in-differences study
Source: PLoS Med. 2019 Jun 18;16(6):e1002821. doi: 10.1371/journal.pmed.1002821 (PMC6581254; doi:10.1371/journal.pmed.1002821)
Supplement: S1 STROBE checklist — (DOCX) [file pmed.1002821.s004.docx]

STROBE Statement—Checklist of items that should be included in reports of ***cohort studies***

|  | Item No | Recommendation | Page number |
| --- | --- | --- | --- |
| **Title and abstract** | 1 | (*a*) Indicate the study’s design with a commonly used term in the title or the abstract | Title page |
|  |  | (*b*) Provide in the abstract an informative and balanced summary of what was done and what was found | Abstract |
| Introduction | | |  |
| Background/rationale | 2 | Explain the scientific background and rationale for the investigation being reported | Introduction, paragraphs 1-3 |
| Objectives | 3 | State specific objectives, including any prespecified hypotheses | Introduction, paragraph 3 |
| Methods | | |  |
| Study design | 4 | Present key elements of study design early in the paper | Abstract; Introduction, paragraph 4 |
| Setting | 5 | Describe the setting, locations, and relevant dates, including periods of recruitment, exposure, follow-up, and data collection | Methods, “Data and Sample” subsection |
| Participants | 6 | (*a*) Give the eligibility criteria, and the sources and methods of selection of participants. Describe methods of follow-up | Methods, “Data and Sample” subsection  S1 Appendix, Pages 1-3 |
|  |  | (*b*) For matched studies, give matching criteria and number of exposed and unexposed | N/A |
| Variables | 7 | Clearly define all outcomes, exposures, predictors, potential confounders, and effect modifiers. Give diagnostic criteria, if applicable | Methods, “Outcomes and Exposure” subsection  Methods “Statistical Analysis” subsection (for predictors and confounders) |
| Data sources/ measurement | 8* | For each variable of interest, give sources of data and details of methods of assessment (measurement). Describe comparability of assessment methods if there is more than one group | Methods, “Data and Sample” and “Outcome and Exposure” subsections  S1 Appendix pages 1-3 |
| Bias | 9 | Describe any efforts to address potential sources of bias | Methods, “Statistical Analysis” and “Sensitivity Analyses” subsections |
| Study size | 10 | Explain how the study size was arrived at | Methods, “Data and Sample” subsections  S1 Appendix, pages 1-3 |
| Quantitative variables | 11 | Explain how quantitative variables were handled in the analyses. If applicable, describe which groupings were chosen and why | Methods, “Data and Sample” and “Statistical Analysis” subsections |
| Statistical methods | 12 | (*a*) Describe all statistical methods, including those used to control for confounding | Methods, “Statistical Analysis” subsection (main analyses)  Methods, “Sensitivity Analyses” subsections (sensitivity analyses)  S1 Appendix, pages 3-6 |
|  |  | (*b*) Describe any methods used to examine subgroups and interactions | Methods, “Statistical Analysis” subsection, paragraph 2 |
|  |  | (*c*) Explain how missing data were addressed | Methods, “Data and Sample” subsection, paragraph 2  S1 Appendix, pages 1-2 |
|  |  | (*d*) If applicable, explain how loss to follow-up was addressed | N/A |
|  |  | (*e*) Describe any sensitivity analyses | Methods, “Sensitivity Analyses” subsection |
| Results | | |  |
| Participants | 13* | (a) Report numbers of individuals at each stage of study—eg numbers potentially eligible, examined for eligibility, confirmed eligible, included in the study, completing follow-up, and analysed | Results, “Descriptive Statistics” subsection  S3 Table |
|  |  | (b) Give reasons for non-participation at each stage | N/A |
|  |  | (c) Consider use of a flow diagram | N/A |
| Descriptive data | 14* | (a) Give characteristics of study participants (eg demographic, clinical, social) and information on exposures and potential confounders | Results, “Descriptive Statistics” subsection  Table 1 |
|  |  | (b) Indicate number of participants with missing data for each variable of interest | S1 Appendix, page 2 and 3 |
|  |  | (c) Summarise follow-up time (eg, average and total amount) | N/A |
| Outcome data | 15* | Report numbers of outcome events or summary measures over time | Table 1 and Table 2 |
| Main results | 16 | (*a*) Give unadjusted estimates and, if applicable, confounder-adjusted estimates and their precision (eg, 95% confidence interval). Make clear which confounders were adjusted for and why they were included | Results, “Difference-in-Differences and Event Study Estimates” subsection (main analyses)  Results, “Sensitivity Analyses” subsection (sensitivity analyses)  Table 2  Figure 2  SI Appendix pages 3-4 |
|  |  | (*b*) Report category boundaries when continuous variables were categorized | N/A |
|  |  | (*c*) If relevant, consider translating estimates of relative risk into absolute risk for a meaningful time period | N/A (we present difference-in-difference estimates of absolute risk throughout) |
| Other analyses | 17 | Report other analyses done—eg analyses of subgroups and interactions, and sensitivity analyses | Results, “Difference-in-Differences and Event Study Estimates” subsection, paragraph 2  Results, “Sensitivity Analyses” subsection  SI Appendix,  S4 Table, S5 Table, S6 Table, S7 Table, S8 Table  S2 Figure |
| Discussion | | |  |
| Key results | 18 | Summarise key results with reference to study objectives | Discussion, paragraph 1 |
| Limitations | 19 | Discuss limitations of the study, taking into account sources of potential bias or imprecision. Discuss both direction and magnitude of any potential bias | Discussion, paragraphs 4-5 |
| Interpretation | 20 | Give a cautious overall interpretation of results considering objectives, limitations, multiplicity of analyses, results from similar studies, and other relevant evidence | Discussion, paragraph 1 and paragraphs 4-5 |
| Generalisability | 21 | Discuss the generalisability (external validity) of the study results | Discussion, paragraph 5 |
| Other information | | |  |
| Funding | 22 | Give the source of funding and the role of the funders for the present study and, if applicable, for the original study on which the present article is based | Financial disclosure statement |

*Give information separately for exposed and unexposed groups.

**Note:** An Explanation and Elaboration article discusses each checklist item and gives methodological background and published examples of transparent reporting. The STROBE checklist is best used in conjunction with this article (freely available on the Web sites of PLoS Medicine at http://www.plosmedicine.org/, Annals of Internal Medicine at http://www.annals.org/, and Epidemiology at http://www.epidem.com/). Information on the STROBE Initiative is available at http://www.strobe-statement.org.
