# Supplementary material for: College affirmative action bans and smoking and alcohol use among underrepresented minority adolescents in the United States: A difference-in-differences study
Source: PLoS Med. 2019 Jun 18;16(6):e1002821. doi: 10.1371/journal.pmed.1002821 (PMC6581254; doi:10.1371/journal.pmed.1002821)
Supplement: S1 Table — (DOCX) [file pmed.1002821.s005.docx]

**S1 Table.** States Implementing College Admission Affirmative Action Bans During Study Period

| **State** | **Year Enacted** | **Source of Ban** | **Vote/Judgement Date** |
| --- | --- | --- | --- |
| Texas^1^ | 1997 | Court Ruling Hopwood v Texas; House Bill 588 | March 18, 1996 |
| California^2^ | 1998 | Voter Initiative Prop 209 | November 5, 1996 |
| Washington^3^ | 1999 | Voter Initiative 200 | November 3, 1998 |
| Florida^4^ | 2001 | Executive Order 99-281 | November 9, 1999 |
| Michigan^5^ | 2006 | Voter Initiative Prop 2 (Civil Rights Amendment) | November 7, 2006 |
| Nebraska^6^ | 2008 | Measure 424 (Civil Rights Initiative 424) | November 4, 2008 |
| Arizona^7^ | 2010 | Voter Initiative Prop 107 | November 2, 2010 |
| N. Hampshire^8^ | 2012 | Legislative Act HB 623 | June 29, 2011 |
| Oklahoma^9^ | 2013 | Voter Initiative State Question 759 | November 6, 2012 |

**Notes:** This table identifies the states that implemented affirmative action bans during the study period (1991-2015), along with the year of enactment/implementation and the legislative or legal instrument used to pass and implement the ban. To build this database, we first reviewed published studies of the socioeconomic impacts of affirmative action bans. We verified (and corrected, if needed) the dates mentioned in these studies by cross-referencing the Westlaw database and by using directed Google searches. We then searched these same databases for implemented affirmative action bans in other states. With the exception of Texas, where some – but not all – colleges and universities reinstituted affirmative action programs after a favorable court decision in 2003, all of the state-level bans listed here remain in force.

1 <http://caselaw.findlaw.com/us-5th-circuit/1222335.html>

2 <http://elections.cdn.sos.ca.gov/sov/1996-general/sov-complete.pdf>

3 <https://www.sos.wa.gov//elections/initiatives/statistics_initleg.aspx>

4 <http://caselaw.findlaw.com/us-5th-circuit/1222335.html>

5 <http://miboecfr.nictusa.com/election/results/06GEN/>

6 <https://web.archive.org/web/20150316183737/http://nebraskalegislature.gov/pdf/bluebook/245-274.pdf>

7 <http://apps.azsos.gov/election/2010/General/Canvass2010GE.pdf>

8 <https://legiscan.com/NH/text/HB623/id/137991>

9 <https://www.ok.gov/elections/support/12gen_seb.html>
