# Supplementary material for: College affirmative action bans and smoking and alcohol use among underrepresented minority adolescents in the United States: A difference-in-differences study
Source: PLoS Med. 2019 Jun 18;16(6):e1002821. doi: 10.1371/journal.pmed.1002821 (PMC6581254; doi:10.1371/journal.pmed.1002821)
Supplement: S2 Table — (DOCX) [file pmed.1002821.s006.docx]

**S2 Table.** YRBS Coverage, by State and by Year

**Notes:** This table describes state-year coverage of the national YRBS. Boldface text denotes states in which an affirmative action ban was implemented at some point during the study period. Shaded areas reflect survey periods coincident with or during the time an affirmative action ban was in place. Of note, the Texas ban was repealed 6 years after it passed (see Table 1).
