# Supplementary material for: College affirmative action bans and smoking and alcohol use among underrepresented minority adolescents in the United States: A difference-in-differences study
Source: PLoS Med. 2019 Jun 18;16(6):e1002821. doi: 10.1371/journal.pmed.1002821 (PMC6581254; doi:10.1371/journal.pmed.1002821)
Supplement: S3 Table — (DOCX) [file pmed.1002821.s007.docx]

**S3 Table**. Exposed and Unexposed Observations by Survey Year and Outcome, YRBS

**Notes:** This table describes the number of observations in the main (underrepresented minority) study sample in each YRBS survey year assigned as being exposed or not exposed to state-level affirmative action bans by study outcome. There are no exposed individuals in the 1991-1995 survey waves because the first affirmative action ban was implemented
