# Supplementary material for: College affirmative action bans and smoking and alcohol use among underrepresented minority adolescents in the United States: A difference-in-differences study
Source: PLoS Med. 2019 Jun 18;16(6):e1002821. doi: 10.1371/journal.pmed.1002821 (PMC6581254; doi:10.1371/journal.pmed.1002821)
Supplement: S4 Table — (DOCX) [file pmed.1002821.s008.docx]

**S4 Table**. Difference-in-Differences Estimates, by Race and Sex

**Notes:** Each panel-column represents a separate regression model fitted to data for the subgroup represented in the panel header. The regression models are identical to those presented in **Table 2** of the main text but are stratified by sex and race/ethnicity. Stratified estimates for Native American respondents are not presented due to small sample sizes. Estimates are presented as percentage point changes, which are computed by taking the linear probability model regression coefficient and multiplying by 100. The 95% confidence intervals corrected for clustering at the state level are shown in square brackets
