# Supplementary material for: College affirmative action bans and smoking and alcohol use among underrepresented minority adolescents in the United States: A difference-in-differences study
Source: PLoS Med. 2019 Jun 18;16(6):e1002821. doi: 10.1371/journal.pmed.1002821 (PMC6581254; doi:10.1371/journal.pmed.1002821)
Supplement: S5 Table — (DOCX) [file pmed.1002821.s009.docx]

**S5 Table.** Sensitivity Analyses for Difference-in-Differences Estimates

**Notes:** This table provides estimates from sensitivity analyses designed to probe the robustness of our estimates to alternative specifications. Column 1 shows the primary estimates, identical to those presented in **Table 2** of the main text. Column 2 displays estimates that are additionally adjusted for state- and year-specific cigarette tax rates, alcohol tax rates, (logarithm of) per capita income, and unemployment rates. Column 3 displays estimates from regression models in which we accounted for potential geographic spillover effects for adolescents in states adjacent to those implementing affirmative action bans, whose college choice set may have included institutions in nearby, ban-implementing states. Specifically, we included in these models a separate binary indicator denoting potential exposure for adolescents in states bordering those implementing affirmative action (in the years these bans were active). Column 4 displays estimates from regression models in which we reclassify as unexposed adolescents living in Texas in 2003 and thereafter. Column 5 displays estimates from regression models fitted to data on respondents only living in states that passed an affirmative action ban at some point during the study period.
