# Supplementary material for: College affirmative action bans and smoking and alcohol use among underrepresented minority adolescents in the United States: A difference-in-differences study
Source: PLoS Med. 2019 Jun 18;16(6):e1002821. doi: 10.1371/journal.pmed.1002821 (PMC6581254; doi:10.1371/journal.pmed.1002821)
Supplement: S6 Table — (DOCX) [file pmed.1002821.s010.docx]

**S6 Table.** Falsification Test for the TUS-CPS

**Notes:** Column 1 presents results from the same specification reported in **Table 2** of the main text. Column 2 presents results from the falsification test: treatment is assigned as being first exposure to affirmative action bans at age 19. This procedure is a falsification test because this age is after the time period during which the majority of individuals make college decisions and, consequently, for whom the ban should not be relevant. As expected, the point estimate on this “placebo” exposure is small in magnitude and not statistically significant.
