# Supplementary material for: College affirmative action bans and smoking and alcohol use among underrepresented minority adolescents in the United States: A difference-in-differences study
Source: PLoS Med. 2019 Jun 18;16(6):e1002821. doi: 10.1371/journal.pmed.1002821 (PMC6581254; doi:10.1371/journal.pmed.1002821)
Supplement: S7 Table — (DOCX) [file pmed.1002821.s011.docx]

**S7 Table.** Analysis of Non-Random Selection Due to High School Dropout and Migration

**Notes:** These estimates are based on regression models fitted to data on under-represented minority (Black, Hispanic, or Native American) individuals aged 16-18 years from the 1991-2015 Current Population Survey Annual Social and Economic Supplement (CPS-ASEC). The CPS-ASEC is conducted annually in March and includes respondents who are both in school and out of school, in contrast to the YRBS (which includes only respondents who are in school). The dependent variables of interest are: whether the individual is currently in school (yes/no) and whether the individual migrated to the current state of residence within the previous year (yes/no). Each column denotes a separate regression model, and the specifications are the same as the regression models displayed in **Table 2** of the main text. The purpose of the CPS-ASEC analysis is to probe for potential bias resulting from selection into the YRBS sample and/or non-random migration (presumably driven by parental relocation decisions), both of which could theoretically be induced by changing affirmative action policies. In general, these null results are consistent with Hinrichs (Rev Econ Stat 2012;94:712-22), who also finds no evidence of non-random migration due to affirmative action policies in a different dataset.
