# Supplementary material for: College affirmative action bans and smoking and alcohol use among underrepresented minority adolescents in the United States: A difference-in-differences study
Source: PLoS Med. 2019 Jun 18;16(6):e1002821. doi: 10.1371/journal.pmed.1002821 (PMC6581254; doi:10.1371/journal.pmed.1002821)
Supplement: S8 Table — (DOCX) [file pmed.1002821.s012.docx]

**S8 Table.** Analyses with and without Sampling Weights

**Notes:** Estimates presented in Column 1 are identical to those in **Table 2** of the main text. In Column 2, we estimated the same regression models but also utilized YRBS and TUS-CPS sampling weights.
